# Exploring the antibacterial properties of honey and its potential

**DOI:** 10.3389/fmicb.2012.00398

**Published:** 2012-11-22

**Authors:** Fernando C. Bizerra, Pedro I. Da Silva, Mirian A. F. Hayashi

**Affiliations:** ^1^Departamento de Medicina, Universidade Federal de São PauloSão Paulo, Brazil; ^2^Centro de Toxinologia Aplicada (CAT-CEPID), Instituto ButantanSão Paulo, Brazil; ^3^Departamento de Farmacologia, Universidade Federal de São Paulo (UNIFESP-EPM)São Paulo, Brazil

**A commentary on**

**Re-examining the role of hydrogen peroxide in bacteriostatic and bactericidal activities of honey**

by Brudzynski, K., Abubaker, K., St-Martin, L., and Castle, A. (2011). Front. Microbiol. 2:213. doi:10.3389/fmicb.2011.00213

In the trend of the use of antimicrobial compounds from natural and renewable resources, natural antimicrobial compounds, particularly found in food and with potential biomedical applications, are of highest interest. In this context, the contribution by Brudzynski et al. to our special issue on “antimicrobial compounds from natural sources” deserves a special attention not only for giving insights into the natural antimicrobial components present in the honey, but also for exploiting their potential mechanism of action.

The antibacterial properties of honey have been well documented. The hydrogen peroxide has been described as the main compound responsible by the antibacterial activity of honeys. The hydrogen peroxide is a potent antimicrobial agent, produced mainly during glucose oxidation catalyzed by the action of the bee enzyme, glucose oxidase, which is introduced into honey during nectar harvesting by bees. The hydrogen peroxide concentration in honey is determined by the rate of its production by glucose oxidase and its destruction by catalases. Thus, the hydrogen peroxide levels in different honeys may differ considerably from honey to honey. In this study the authors re-examined the role of the hydrogen peroxide as component responsible for the antibacterial activity in honey.

The correlation between the endogenous hydrogen peroxide concentration and the inhibitory activity of bacterial growth by honey is well established. Indeed, honeys with a high concentration of hydrogen peroxide have higher antibacterial activity. However, honey is a complex chemical milieu composed of over 100 different compounds (including antioxidants and traces of transition metals), which can interact with the hydrogen peroxide, affecting the oxidizing activity of the honey. Consequently, this interaction may result in increase or decrease of the antimicrobial activity of honey.

Hydrogen peroxide alone is commonly used as disinfectant compound in medical equipment in hospitals. For disinfection of these materials high concentrations of H_2_O_2_ are used (0.8–8 M) and H_2_O_2_ antimicrobial activity has been verified against several medical important bacteria species, including *Staphylococcus* spp., *Streptococcus* spp., *Pseudomonas* spp. and *Bacillus* spores. The bactericide activity of hydrogen peroxide is related to the accumulation of irreversible oxidative damages to the membrane, proteins, enzymes, and DNA.

The hydrogen peroxide content in honey is about 900-fold lower than the concentration commonly used for disinfecting medical equipments. *In vitro* studies showed that the cell death of cultured mammalian, yeast and bacterial cells is observed with higher than 50 mM of H_2_O_2_ and was associated with chromosomal DNA degradation. However, this concentration is still about 50-fold higher than the concentration of hydrogen peroxide found in honey.

The authors hypothesized that the oxidizing action of honey's hydrogen peroxide on bacterial cells may be modulated by the presence of other bioactive molecules in honey and therefore, may differ from the action of hydrogen peroxide alone. Thus, the aim of the study was to critically analyze the effects of hydrogen peroxide on growth and survival of bacterial cells in order to analyze its putative role as a main component responsible for the antibacterial activity of honey.

Several natural and artificial honey samples were evaluated by the authors, using *Bacillus subtilis* and *Escherichia coli* as bacterial reference strains, to determine the correlation between the hydrogen peroxide concentration and the bacterial growth inhibition ability. Moreover, the treatment of honey samples with catalase allowed verifying the effects of the inhibition of oxidizing activity of the endogenous hydrogen peroxide on antibacterial activity and on DNA degradation of the bacterial cells.

The antibacterial susceptibility tests with exogenous H_2_O_2_ showed a concentration-dependent inhibition of *E. coli* and *B. subtilis* growth of about 90% at 1.25 mM and 2.5 mM, respectively. However, among the different honeys samples analyzed in this study, the concentration of hydrogen peroxide ranged from 0.248 ± 0.02 to 2.68 ± 0.04 mM. Endogenous H_2_O_2_ found in Canadian honeys inhibited the growth of *E. coli* in a concentration-dependent manner, and its MIC_90_ was two-fold higher compared to that determined for exogenous H_2_O_2_. In contrast, honey H_2_O_2_ was ineffective to inhibit *B. subtilis* growth, suggesting that other honey compounds or physical features, such as high osmolarity, were responsible for the antimicrobial properties of honeys. Paradoxical growth stimulation of *B. subtilis* was also observed at high honey dilutions (16-fold and over) and in the presence of high levels of H_2_O_2_. According to the authors, these differences could be related to difference in the bacterial susceptibility profile to the oxidative action of hydrogen peroxide and/or interference from other honey compounds.

Little is known about the role of the honey's hydrogen peroxide to bacterial cell death. Considering that the H_2_O_2_ triggered cell death of cultured mammalian, yeast, and bacterial cells was associated with chromosomal DNA degrades, the authors also examined the action of honey H_2_O_2_ in bacterial DNA degradation, which is a lethal event that ultimately kills the cell. In general, despite the presence of antioxidants in honey such as catalases, polyphenols, Maillard reaction products, and ascorbic acid that would lower the oxidative stress to cells, DNA degradation of *E. coli* cells exposed to exogenous or endogenous H_2_O_2_. In fact, the lower concentrations of honeys H_2_O_2_ required to effectively degrade chromosomal DNA led the authors to suggest that the oxidizing effect of H_2_O_2_ was augmented by other honey components such as transition metals (Fe, Cu) also commonly present in honeys.

In conclusion, this study represents an important step in understanding the antibacterial properties of honey. The findings described in this study not only revise the old views but also provide novel information on the role of hydrogen peroxide in the regulation of bacteriostatic and bactericidal activities of honey. The identification of the main components responsible for the antibacterial activity of honey and the mechanism of action underlying this effect bring new knowledge to the field that will greatly contribute to understand the mechanisms by which honey compounds lead to bacterial growth inhibition and bacterial death.

